# Polymerase chain reaction for the diagnosis of herpesvirus infections in dermatology: Analysis of clinical data

**DOI:** 10.1007/s00508-019-01585-w

**Published:** 2019-12-09

**Authors:** Verena Schremser, Lukasz Antoniewicz, Erwin Tschachler, Alexandra Geusau

**Affiliations:** 1 Department of Dermatology, Medical University of Vienna, Waehringer Guertel 18-20, 1090 Vienna, Austria; 2 Dept. of Clinical Sciences, Karolinska Institutet at Danderyds University Hospital, Stockholm, Sweden; 3 Research Department of Biology and Pathobiology of the Skin, Medical University of Vienna, Vienna, Austria

**Keywords:** Polymerase chain reaction, Virology, Herpetic skin manifestations, Herpes simplex, Immunosuppression

## Abstract

**Background:**

Rapid identification of human herpesviruses from lesion swabs is necessary for timely initiation of antiviral treatment, especially with infections involving neonates and immunocompromised individuals. The aim of the study was to investigate the results of an in-house polymerase chain reaction (PCR) test for herpesviruses in patients with symptoms suggestive for a herpesvirus infection.

**Patients and methods:**

In this single center retrospective study the results of 3677 lesion swab specimens tested for human herpes simplex virus 1 and 2 (HSV 1 and 2) and varicella zoster virus (VZV) were analyzed in the context of data sheets giving details of the suspected diagnosis, medical history as well as the demographic data of the patients. The PCR procedures for cytomegalovirus (CMV), Epstein-Barr virus (EBV) and human herpes virus 8 (HHV-8) were applied on special occasions.

**Results:**

Of the samples 3369 (91.6%) were swabs and a minority were tissue or blood samples. Of the 3015 samples tested for HSV‑1, HSV‑2 and VZV concomitantly, 52.3% were positive for at least one of these viruses. Clinically distinct conditions, such as herpes zoster and varicella had a high rate of positive PCR results, ranging from 81% to 88%, respectively. Among HSV‑2 positive samples, 23.7% derived from human immunodeficiency virus (HIV) positive patients, in contrast to the 10.8% originating from immunocompetent patients, the difference being statistically significant (*p* < 0.002). The HSV‑2 was detected more often in women than in men.

**Conclusion:**

Distinct clinical diagnoses have a high correlation rate with positive PCR results. A significantly higher number of HSV‑2 positive results were found in HIV positive patients and in women.

## Introduction

Human herpesvirus (HHV) infections are common worldwide. Clinically, herpes simplex virus 1 and 2 (HSV‑1 and HSV‑2, respectively) and varicella zoster virus (VZV) result in a range of diseases involving the skin and mucous membranes, occasionally leading to severe complications, such as herpetic neuralgia and herpes encephalitis. Due to similarities in the clinical presentations of infections caused by these three herpesviruses, a clinical diagnosis needs to be confirmed by laboratory testing and direct proof of the pathogen to prevent misdiagnosis and a delay in the initiation of appropriate antiviral treatment. Therefore, early diagnosis with a highly sensitive method is mandatory [1]. Traditional laboratory methods for the diagnosis of cutaneous and mucocutaneous HSV‑1, HSV‑2, and VZV infections include the Tzanck smear, direct fluorescent assay (DFA), and cell culture. Although viral culture has been considered the gold standard test, several reports have been published on the much higher sensitivity of polymerase chain reaction (PCR) assays for detecting HSV and VZV over the traditional methods [2–6]. In the year 2000 when a herpes PCR test was not yet commercially available, an in-house PCR amplification technique for the detection of HSV‑1, HSV‑2, VZV as well as other members of the herpesvirus family, such as cytomegalovirus (CMV), Epstein-Barr virus (EBV) and human herpesvirus 8 (HHV-8), were established at the Department of Dermatology, Medical University of Vienna and further employed on a routine level for samples directly acquired from active lesions.

The purpose of this retrospective study was to analyze the correlation of the PCR results and the clinical diagnoses supplied on standardized data sheets giving details on individual-related facts, medical history and demographic data of the patients. Furthermore, the study aimed to depict the distribution of the various herpesviruses detected in different patient populations with different clinical diagnoses.

## Material and methods

### Study population and data collection

In this single center study, test results of swab samples taken from presumably herpesvirus-associated lesions on the skin and mucous membranes which had been analyzed by a routine PCR testing for HSV‑1, HSV‑2 and VZV, were retrospectively evaluated at the Department of Dermatology, Medical University of Vienna. An in-house herpes PCR technique was performed on a total of 3677 samples collected during a period of 4 years. A standardized data sheet was compiled of each case giving details of the suspected diagnosis, sample site, the test requested as well as demographic data of the patients including age, gender and immune status. Several types of viral genome were investigated in each sample concomitantly, in particular HSV 1 and 2, VZV, whereas CMV, EBV and HHV‑8 only when requested by the clinician. The results of the PCR analysis documented on these data sheets were anonymized for this analysis. Missing data were collected from archived patient charts. Finally, the clinical diagnoses were categorized based on similarities of the clinical manifestation mentioned on the order form. The data were then transferred to SPSS (Statistical Package for the Social Sciences, IBM® SPSS Statistics®, version 24, Chicago, IL, USA).

#### Sample and specimen collection:

Using a sterile cotton tip or disposable scalpel, swabs from vesicles, pustules, erosions on skin and mucous membranes were sampled after gently removing the roof of the vesicle or pustule in order to collect the fluid and cellular material at the base of the lesion. If the site was ulcerated, crusts of the lesions were removed. The obtained material was applied on a simple slide and passed to the laboratory. The DNA from biopsy samples was transferred to the laboratory in sterile tubes.

#### DNA extraction:

From the slides, the material was scraped off and transferred to sterile containers suspended in 1.0 ml sterile H_2_O, boiled for 15 min and stored at + 4 C. For all samples, DNA was extracted using a DNA extraction kit (Qiagen DNeasy blood and tissue kit [QIAGEN, Hildesheim, Germany]), a procedure carried out according to the manufacturer’s instruction.

#### PCR amplification for HSV1, HSV2, VZV, B-actin:

The amplification reaction was carried out in 50 µl reaction mixture, containing 5 µl of 10X PCR reaction buffer, 1 µl of 10 mM dNTPs, 3 µl MgCl_2_, 2.5 µl of 10 pmol forward primers, 2.5 µl of 10 pmol reverse primers, 0.1 µl of 5 U/µl Red hot Taq deoxyribonucleic acid (DNA) polymerase (Thermo Scientific, Thermo Fisher Scientific, Inc., Waltham, MA, USA) and 5 µl DNA samples (100 ng). Amplification was carried out with a thermal profile at 94 °C for 4 min initial denaturation then 40 cycles (94 °C for 1 min, 58 °C for 30 s and 72 °C for 1.5 min) amplification with a final extension of 72°C for 8 min. The primer pair (B-actin‑s and B‑actin-as) was used to amplify (570 base pair) human housekeeping gene B‑actin, the primer pair (HSV1‑s and HSV1-as) was used to amplify (448 base pair) HSV1 DNA, the primer pair (HSV2‑s and HSV2-as) was used to amplify (239 base pair) HSV2 DNA, the primer pair (VZV‑s and VZV-as) was used to amplify (326 base pair) fragment of VZV DNA.

#### PCR amplification for HV8, CMV and EBV:

The amplification reaction was performed as described above. Amplification was carried out with a thermal profile at 94 °C for 4 min initial denaturation then 45 cycles (94°C for 1 min, 58°C for 30 s and 72°C for 1.5 min) amplification with a final extension of 72°C for 8 min. The primer pair (HV8‑s and HV8-as) was used to amplify (335 base pair) HV8 DNA, the primer pair (CMV‑s and CMV-as) was used to amplify (453 base pair) fragment of CMV DNA, the primer pair (EBV‑s and EBV-as) was used to amplify (200 base pare) fragment of EBV DNA.

The primer pairs that are illustrated in the following table (B-actin‑s and B‑actin-as, HSV1‑s and HSV-1as, HSV2‑s and HSV2-as, VZV‑s and VZV-as, HV8‑s and HV8-as, CMV‑s, CMV-as, EBV‑s and EBV-as) were used to amplify the requested herpes virus DNA (Table 1).

**Table 1:** Primers used in the amplification of different human herpesviruses

Name	Sequences 5′–3′
HSV1‑s	CAA CTA CCC CGA TCA TCA GTT A
HSV1-as	ACA GTT GCC TCC CAT CCG AAA CCA A
HSV2‑s	CAT CGG CGG TAT TGC GTT TTG GGT A
HSV2-AS	CCT ATG AAC TGT CCT AGT TTC C
VZV‑s	GCT CGT TGA GGA CAT CAA CCG TGT T
VZV-as	CAT CGT CGC TAT CGT CTT CAC CAC
EBV‑s	ACG AGG GGC CAG GTA CAG GA
EBV-as	CAC CAT CTC TAT GTC TTG GC
CMV‑s	CAG CAC CAT CCT CCT CTT CCT CTG G
CMV-as	CCA AGC GGC CTC TGA TAA CCA AGC C
HV8‑s	ATG GAT CCC TCT GAC AAC CTT
HV8-as	CGT GGA TCC GTG TTG TCT ACG
BA‑s ^a^	TTC CCC TCC ATC GTG GGG CGC CCC AGG CAC CAG GGC
BA-as	GGC GAC GTA GCA CAG CTT CTC

*BA* beta actin, *HSV1* Herpes simplex virus 1, *HSV2* Herpes simplex virus 2, *VZV* Varicella zoster virus, *EBV* Epstein-Barr virus, *CMV* Cytomegalovirus, *HV8* Human herpesvirus 8

^a^primer for the amplification of beta actin DNA

### Statistical analyses

The statistical analysis was performed using SPSS 21.0 software for windows (SPSS Inc., Chicago, IL, USA). Metric data were described by mean values and standard deviations. Nonmetric data, namely clinical diagnosis, localization, immune status and sex were described by contingency tables and frequency analysis. Appropriate graphs were used to illustrate both types of variables. For the qualitative data analysis χ^2^-tests or Fisher’s exact tests were used. All *p*-values were two tailed and considered statistically significant at <0.005.

## Results

### Patients and samples

Basic characteristics of patients and samples are listed in (Table 2).

**Table 2:** Basic characteristics of patients and samples

**Number of patients**	**3202**
*Gender*	
Male	1605 (50.1%)
Female	1597 (49.9%)
*Age (years)*	
Average ± SD	45.6 ± 20.7
Range	0–99
**Number of samples**	**3677**
*Samples derived from*	
Immunocompetent patients	3146 (85.6%)
HIV-positive patients	338 (9.2%)
Patients under immunosuppressive drugs	71 (1.9%)
Bone marrow transplant patients	67 (1.8%)
Solid organ transplant recipients	55 (1.5%)
*Sample material*	
Swabs	3369 (91.6%)
Blood sample	203 (5.5%)
Rinsing liquid	69 (1.9%
Tissue	35 (1.0%)
Liquid aspirate	1 (0.0036%)
*Sample sites*	
Genital area	647 (17.6%)
Head and neck area	437 (11.9%)
Oral mucosa	435 (11.8%)
Lips	393 (10.7%)
Trunk	307 (8.3%)
Blood	203 (5.5%)
Sacral/gluteal	202 (5.5%)
Eye region	176 (4.8%)
Lower limbs	161 (4.4%)
Upper limbs	146 (4.0%)
Abdomen	79 (2.1%)
Flank	49 (1.3%)
Fingers	35 (1.0%)
Inguinal region	23 (0.6%)
Not known	362 (9.8%)
Others	22 (0.6%)

*HIV* Human Immunodeficiency Virus

Altogether 3677 specimens were taken from 3202 patients, who had an average age of 45.6 years (±20.7 years); 1597 of the specimens derived from female and 1605 from male patients. In 14.4% (*n* = 531) of the cases, the specimens came from immunocompromised patients, i.e. 338 (9.2%) of the samples had been taken from human immunodeficiency virus (HIV) infected individuals, 71 (1.9%) from patients under immunosuppressive therapy due to various reasons, 67 (1.8%) from bone marrow and 55 samples (1.5%) from solid organ transplanted patients.

Sample material was mainly swabs (91.6%), 5.5% were blood samples, the remaining were rinsing liquid, paraffin embedded or native tissue samples and aspirates. In 17.6% the genital area was the sample site, in 11.9% and 11.8%, respectively, samples derived from the head/neck area and oral mucosa, followed by the lips (10.7%) and the eye-region (4.8%). Further samples originated from the area of the trunk (8.3%), the sacrum (5.5%), the abdomen (2.1%), the flank (1.3%), upper limbs (4.0%) and lower limbs (4.4%). A small proportion of the samples derived from the fingers and the inguinal region. In 9.8% the sample site was not listed in the files.

In clinical practice at the dermatology department, swabs from herpetiform lesions from the skin and mucous membranes, were not only analyzed for HSV‑1 and HSV‑2, but the analysis was routinely combined with that for VZV. This was the case for 3015 samples out of the 3677 specimen, which is referred to as testing for the triplet HSV‑1, HSV‑2 and VZV. Half of the samples tested for the triplet were tested negative for all three viruses (*n* = 1437, 47.7%). In 18.2% of the tested samples (*n* = 550) HSV‑1 was verified, in 11.3% (*n* = 341) HSV‑2 and in 22.8% (*n* = 687) VZV was detected.

### Suspected clinical diagnoses in association with the test results

**Fig. 1:**
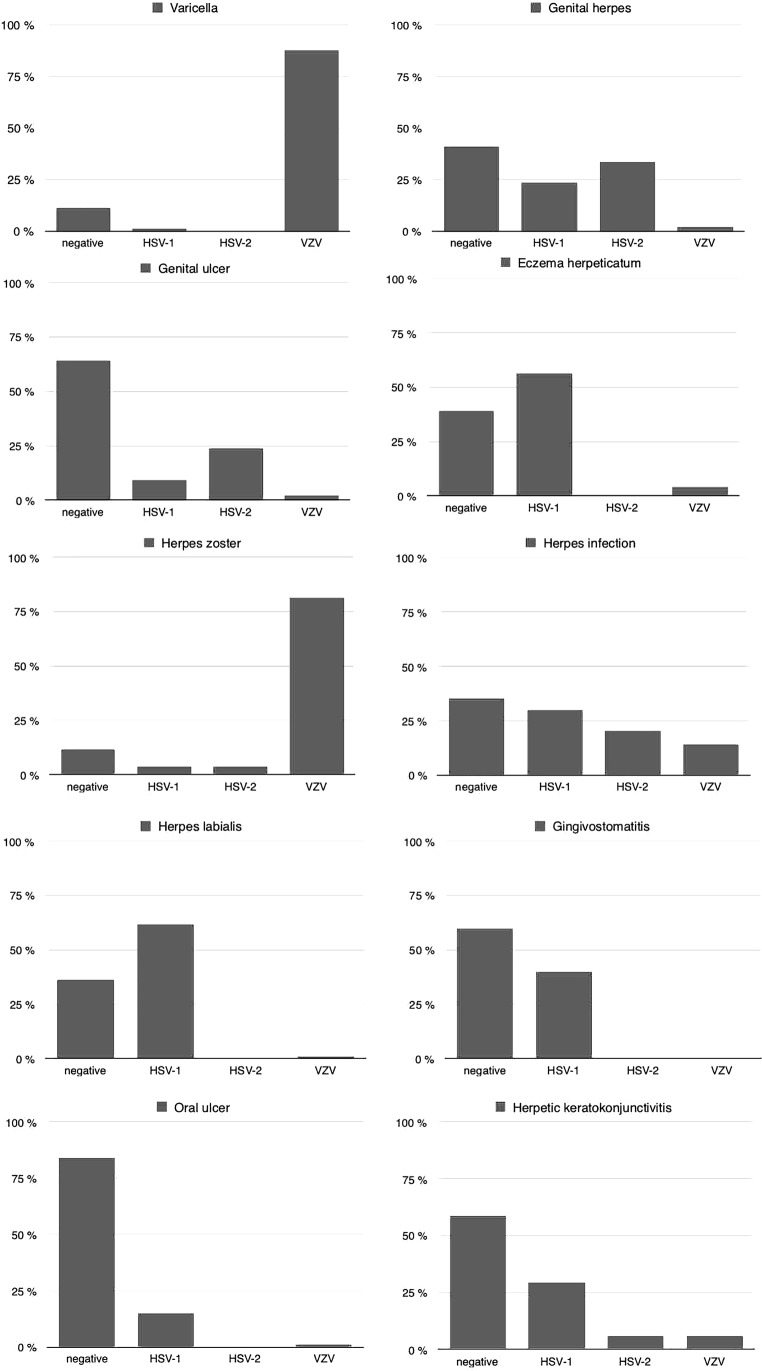
PCR results for the triplet (HSV‑1, HSV‑2, VZV) for the different clinical diagnoses categorized based on similarities of the clinical manifestation mentioned on the order form

A high correlation rate was found between the clinical diagnosis of varicella (87.5%) and the proof of the suitable causative agent, namely VZV. In those cases where explicitly genital, gluteal or recurrent genital herpes was cited in the charts, the investigation gave a positive PCR result in 59.1%, either for HSV‑1 (23.7%) or HSV‑2 (33.3%) or VZV (2.1%). A similar constellation was observed for a similar clinical diagnosis given in the order form, namely genital ulcer in 42 patients, which led to a positive PCR result in 35.7% of the cases, for HSV‑1 in 9.5%, for HSV‑2 in 23.8% and for VZV 2.4% (for details see Fig. 1). The 42 swabs derived from 21 women and 21 men and positive PCR results were more often found in women (57.9%), than men (42.1%). The majority (68.4%) of the positive results derived from immunocompetent patients, whereas 31.6% were immunosuppressed due to various reasons (10.5% HIV, 5.3% bone marrow transplant, 15.8% organ transplant), 7 patients with genital ulcer disease, negative for the triplet, were additionally tested for EBV and 23 for CMV and for each of those viruses, 2 patients tested positive.

The diagnosis of eczema herpeticatum was supported by the proof of HSV‑1 from a lesion swab in 56.5%; no samples tested positive for HSV‑2 in this scenario, whereas a small percentage (4.3%) were positive for VZV.

The clinical diagnosis of herpes zoster corresponded well with the proof of VZV in 81.3% of the cases. When the clinical investigator had sent samples from any site of a clinically herpetic lesion, excluding the genital or oral region, specified with the diagnosis herpes infection, a positive result was achieved for HSV‑1 in 30.0%, for HSV‑2 in 20.6% and for VZV in 14.1%, with a total of 64.7% positive results. The classical diagnosis of labial herpes was supported by an exclusively positive PCR for HSV‑1 in 61.9%, the same applied for the scenario where the clinicians stated (aphthous) gingivostomatitis or oral ulcer, corresponding to positive PCR results for HSV‑1 in 40.0% and 15.0%, respectively, but no HSV‑2 and VZV could be isolated only on one occasion. Eventually these samples derived from patients with primary HSV‑1 infections. Swabs from lesions with the diagnosis herpetic keratoconjunctivitis led to a positive PCR result in 41.2%; in 29.4% HSV‑1, in 5.9% HSV‑2 and in 5.9% VZV were isolated.

With respect to the PCR results for HSV‑2 in general (either positive or negative), an equal male to female ratio was observed in this cohort, whereas there was a shift of the ratio to 1:1.3 towards the female population in those who tested positive for HSV‑2, regardless of the sample site or the stated diagnosis. This result was borderline statistically significant (χ^2^-test; *p* = 0.042, data not shown). Apart from that 23.7% of all HSV‑2 positive results were found in samples from HIV positive individuals, whereas only 10.8% of the samples originated from immunocompetent patients. This difference was statistically significant (*p* < 0.002). Among the patients with a positive PCR result for one of the viruses of the triplet, a male to female ratio of 4:6 was observed and one third (31.6%) of the patients were immunocompromised, 10.5% of them being medically immunosuppressed, 15.8% HIV positive and 5.3% bone marrow transplanted.

## Discussion

Over a 4-year period 3677 mostly cutaneous and mucocutaneous specimens from patients were submitted for testing using an in-house PCR. A high correlation between the suspected clinical diagnosis and the PCR result was found, especially for skin manifestations with distinct clinical manifestations compatible with herpetic pathogenesis. Diagnoses such as cold sore, shingles or chickenpox had a high correlation rate with a positive PCR result ranging from 60% to 88%. A significant difference was found between the HSV‑2 detection rate in the HIV positive and the HIV negative cohort regardless of the sample site, as the HSV‑2 detection rate was two times higher in the HIV positive patient. This observation is supported by a number of other publications [7–9]. Genital herpes has played a more important role than any other sexually transmitted infection in driving HIV prevalence not only in Africa [10, 11]. As the herpes simplex virus breaks down the epithelium, these microdisruptions serve as a portal for the entry of HIV. Additionally, HSV‑2 infected epithelial cells secrete cytokines, which attract immune cells such as dendritic and Langerhans cells. These cells serve as targets and promote the acquisition of HIV [12, 13]. Previous studies have shown that people suffering from HIV and recurrent genital herpes with HSV‑2 reactivation, have a higher HIV transmission rate [14].

In this study population, HSV‑2 could be significantly more frequently detected in women than in men (1.3:1). This finding is supported by similar observations by other investigators [15, 16], e.g. Looker et al. reported a female to male ratio of 1.8:1 [17]. This difference might be explained by female sex hormones being risk factors for the acquisition of sexually transmitted viral diseases, such as HSV‑2 in an animal model [18].

There were 42 patients presenting with the clinical diagnosis genital ulcer disease, characterized by a single ulcer bigger than a typical herpetic skin lesion. Only 35.7% of the samples obtained from these lesions tested positive for HSV‑1, HSV‑2 or VZV, where HSV‑1 was detected in 4, HSV‑2 in 10 patients and VZV in 1 patient; in 2 patients CMV and another 2 EBV was proven. The high number of negative PCR results indicated that the majority of genital ulcer diseases were due to other reasons, including infection with *Treponema pallidum*, non-infectious diseases such as autoimmune bullous diseases, Behcet’s disease or fixed drug eruptions [19]. In this study 10.5% of the patients with genital ulcer disease due to herpes virus infection were HIV positive, equally distributed among males and females, other studies showed higher HIV rates ranging from 27% to 40% [20, 21]. The relatively low number found in this study indicates that the study population represents an average common population, rather than a subset of individuals with high risk sexual behavior.

The in-house PCR method with simply applying material onto a slide is robust, not being influenced by transport or storage and supplying a sufficient amount of viral DNA. Therefore, the method could be an option in low-budget developing countries.

In summary, distinct clinical diagnoses have a high correlation rate with a positive PCR result. A significantly higher rate of HSV‑2 positive results was found in HIV positive patients and women. The data illustrate that the direct proof of herpes viruses has a high diagnostic value, especially in daily clinical life.
